# Impact of the delay in cryopreservation timing during biobanking procedures on human liver tissue metabolomics

**DOI:** 10.1371/journal.pone.0304405

**Published:** 2024-06-10

**Authors:** Corentine Goossens, Vincent Tambay, Valérie-Ann Raymond, Louise Rousseau, Simon Turcotte, Marc Bilodeau

**Affiliations:** 1 Laboratoire d’Hépatologie Cellulaire, Centre de Recherche du Centre Hospitalier de l’Université de Montréal (CRCHUM), Montréal, QC, Canada; 2 Biobanque et Base de Données Hépatopancréatobiliaire, Centre Hospitalier de l’Université de Montréal (CHUM), Montréal, QC, Canada; 3 Département de Chirurgie, Service de Transplantation Hépatique et de Chirurgie Hépatopancréatobiliaire, Centre Hospitalier de l’Université de Montréal (CHUM), Montréal, QC, Canada; 4 Département de Médecine, Université de Montréal, Montréal, QC, Canada; University of California Riverside, UNITED STATES

## Abstract

The liver is a highly specialized organ involved in regulating systemic metabolism. Understanding metabolic reprogramming of liver disease is key in discovering clinical biomarkers, which relies on robust tissue biobanks. However, sample collection and storage procedures pose a threat to obtaining reliable results, as metabolic alterations may occur during sample handling. This study aimed to elucidate the impact of pre-analytical delay during liver resection surgery on liver tissue metabolomics. Patients were enrolled for liver resection during which normal tissue was collected and snap-frozen at three timepoints: before transection, after transection, and after analysis in Pathology. Metabolomics analyses were performed using ^1^H Nuclear Magnetic Resonance (NMR) and Liquid Chromatography-Mass Spectrometry (LC-MS). Time at cryopreservation was the principal variable contributing to differences between liver specimen metabolomes, which superseded even interindividual variability. NMR revealed global changes in the abundance of an array of metabolites, namely a decrease in most metabolites and an increase in β-glucose and lactate. LC-MS revealed that succinate, alanine, glutamine, arginine, leucine, glycerol-3-phosphate, lactate, AMP, glutathione, and NADP were enhanced during cryopreservation delay (all p<0.05), whereas aspartate, iso(citrate), ADP, and ATP, decreased (all p<0.05). Cryopreservation delays occurring during liver tissue biobanking significantly alter an array of metabolites. Indeed, such alterations compromise the integrity of metabolomic data from liver specimens, underlining the importance of standardized protocols for tissue biobanking in hepatology.

## Introduction

The liver is a major metabolic organ and is a hub for systemic metabolism. Diverse metabolic pathways and adaptive responses are managed by the liver. Within the liver, regional differences in metabolic activity are known to take place and have given raise to the concept of liver zonation [1, 2]. The liver plays an important role in energy metabolism by regulating glycogen and glucose production and breakdown as well as those of lipids and fatty acids, and is an important site for protein synthesis [1–3]. The main site of xenobiotic detoxication is the liver, which is also the heart of ammonia elimination through the urea cycle [1, 2, 4].

The metabolic activity of the liver is highly adaptable to changes in physiological or pathological conditions. For example, hepatic lipolysis and fatty acid oxidation, which result in the synthesis of acetyl-CoA for ketone body synthesis, is an adaptive mechanism for systemic energy production during fasting [3, 5]. During liver regeneration, hepatocellular proliferation is dependent on systemic and hepatic changes in metabolism, such as an increase in hepatic fatty acid uptake [6, 7]. Ischemia has also been shown to lead to a decrease in oxidative phosphorylation and subsequent ATP synthesis, an increase in anaerobic metabolism, and cellular acidosis in hepatocytes [8]. Liver metabolism is also altered in various diseases such as metabolic syndrome, type 2 diabetes mellitus, and cancer [5]. Thus, studying liver metabolism is of predominant interest in the pursuit of our understanding of metabolic changes that occur during physiological and pathological conditions. There is currently a major effort to identify novel metabolic biomarkers, which could lead to improve patient care and treatment specificity [9, 10]. The discovery of robust and clinically relevant biomarkers remains one of the main challenges for the early detection, prevention, and treatment of liver diseases. With the advent of high-throughput techniques, metabolomics has the potential to allow for a comprehensive overview of all metabolic activities and biochemical events that occur in liver tissue. To do so, liver tissue biobanks offer a matrix for the identification of metabolic biomarkers of liver diseases and for studying the metabolic activities of normal and diseased liver.

In the context of biobanking, liver specimens must closely reflect the reality of *in situ* tissue for subsequent metabolomics analyses if one aims to obtain the actual metabolome of the liver *in vivo*. As such, quality of biobanking and biospecimen is a critical issue: the diversity of metabolites as well as the complex and unstable nature of tissue samples can easily compromise the acquisition of systematic and reproducible results [11]. Given the adaptive and flexible nature of hepatocyte metabolism, unstandardized procedures for liver tissue biobanking pose a threat to the validity and accuracy of subsequent metabolomics analyses. A major source of biobank specimens are surgical procedures. It is recognized that surgical procedures themselves with their inherent warm and cold ischemia are important parameters to consider for metabolomics [12]. Surgical specimens, in addition to liver biopsies, a key technique for liver tissue collection, are often kept at room temperature or on ice until pathological examination, before being stored in liquid nitrogen. Yet, continuous enzymatic and cellular adaptations can alter their original metabolic profile and therefore compromise the biological interpretation and exactness of metabolomics studies. External factors such as freeze/thaw cycles and inevitable delays in sample collection and freezing significantly impact the quality of collected specimens [13–17]. Therefore, the impact of external factors such as preanalytical handling and methodology of sampling must be minimized and/or standardized. Standard operating procedures have thus been proposed to obtain reliable and reproducible results [18–21]. However, standard protocols for the collection of human tissue specimens remain to be established for metabolomics studies. Herein, this study aimed to characterize the impact of cryopreservation delays of liver samples during resection on tissue metabolomics. Identifying metabolomic alterations that occur during sample collection will draw attention to the importance of standardized liver tissue biobanking protocols, which will help improve procedures for proper and accurate interpretation of metabolomic data obtained from liver.

## Materials and methods

### Patients and tissue collection

Liver specimens were obtained from patients (n = 5) undergoing liver surgery, collected and cryopreserved at three different steps. The first samples were collected before liver transection (T1) and snap-frozen in liquid nitrogen immediately in the operating room. The second (T2) samples were obtained at the moment liver specimens were removed from the abdominal cavity and snap-frozen. The third samples (T3) were snap-frozen after transport of the surgical specimens to the Pathology laboratory and standardized macroscopic pathological examination. Each time point was timed between skin incision (T0) and freezing of the specimen. Activities of the Bank of Blood and Tissue Samples on colorectal, hepatobiliary and pancreatic cancers were carried out in collaboration with the Cancer Research Network (Fonds de recherche du Québec-Santé) affiliated with the Canadian Network of Tissue Banks (CBCN). This research protocol conformed to the Declarations of Helsinki and Istanbul and was approved by the “Comité d’éthique du CRCHUM”, Montréal, Canada. Written informed consent was obtained from all participants prior to surgery. Patients were recruited from 05/10/2018 through 08/14/2018; samples and data were collected on 08/17/2018. Authors had no access to information that could identify individual participants during or after data collection.

### Mass spectrometry sample preparation

Water-soluble metabolites were extracted from liver samples by liquid-liquid extraction and concentrated before LC-MS/MS analysis. Briefly, samples (20 mg) were homogenized in ice-cold extraction buffer (80% methanol, 2 mM ammonium acetate, pH 9, containing 20 μM AMP-^13^C_10_,^15^N_5_ as an internal standard; 30 μL/mg of tissue) using a Cryolis-cooled Precellys 24 Dual system (Bertin, France) with CK14 ceramic beads, 2 x 25 s at 6000 rpm with a 15 s pause. After centrifugation (10 min, 20000g, 4°C), 183 μL of supernatants were transferred to 10 x 75 mm glass tubes, and 367 μL of extraction buffer was added to each tube. Diluted samples were mixed and incubated on ice for 10 min; then, 250 μL of water and 880 μL of chloroform:heptane (3:1) were added and samples were mixed thoroughly and incubated for 15 min on ice. Samples were centrifuged for 15 min (4500g, 4°C): 500 μL of the aqueous phase was transferred to polypropylene tubes for concentration. Organic solvents were removed in a refrigerated CentriVap (Labconco, USA; 90 min at 10°C) and the remaining liquid (100 μL) was freeze-dried overnight. Dry samples were stored at -80°C until they were resuspended in 40 μL of water prior to LC-MS analysis and centrifuged at maximum speed for 5 min at 4°C. Supernatants were transferred to HPLC vials.

### Mass spectrometry analysis

Chromatographic separation of samples and standard solutions was performed using a Nexera X2 Ultra-HPLC system (Shimadzu, Japan) at 40°C with 3 μL injections on a Poroshell 120 EC-C18 2.1 x 75 mm x 2.7 μm Ultra-HPLC column following a Poroshell 120 EC-C18 2.1 x 5 mm x 2.7 μm UHPLC guard column (Agilent Technologies, USA), using gradient elution with an initial mobile phase consisting of 95% solution A (10 mM tributylamine, 15 mM acetic acid in water, pH 5.2) and 5% solution B (acetonitrile:H_2_O (95:5, v/v) fortified with 0.1% formic acid) at a flow rate of 0.75 mL/min. Analytes were detected using a SCIEX 4000 Qtrap mass spectrometer (Framingham, USA) operated in negative electrospray ionization mode. MS/MS parameters were optimized for each metabolite and quantified using MultiQuant 3.0.2 (SCIEX) based on calibration curves (from 0.15 to 12000 pmol per injection) prepared in water from high-purity chemicals (Sigma, USA). Values were normalized per mg of wet tissue.

### Nuclear Magnetic Resonance (NMR) sample preparation

Metabolite extraction was performed from 25 mg of frozen tissue using a dual-phase methanol:H_2_O:chloroform (2:1:2) extraction procedure. Tissues were ground and homogenized using a Precellys Homogenizer (Bertin instruments, France) in 600 μL of methanol/H_2_O solution (2:1 v/v) containing ceramic beads, after which 400 μL of chloroform was added. Samples were incubated at 4°C for 15 min, then centrifuged for 15 min at 15000g and 4°C. The upper polar phase (600 μL) was collected and dried overnight under nitrogen flow. For NMR analysis, dried tissue extracts were dissolved in 600 μL of D_2_O-prepared phosphate buffer (pH 7.4) containing 0.4 mM of sodium trimethylsilyl-(2,2,3,3-d_4_)-propionate (TSP) as an internal reference; 580 μL of the mixture was transferred into 5 mm NMR tubes (Wilmad-LabGlass, USA).

### Nuclear Magnetic Resonance analysis

^1^H NMR analysis was performed on a Bruker Ascend 700 MHz spectrometer (Massachusetts, USA) coupled to a Bruker AVANCE NEO console equipped with a 5 mm triple resonance probe at 298 K. For each sample, one-dimensional NMR spectrum was acquired with water peak suppression using a nuclear overhauser enhancement spectroscopy pre-saturation pulse sequence, 128 scans, 65k data points, an acquisition time of 2.4 s, a relaxation delay of 4 s, a mixing time of 10 ms and a spectral width of 20 ppm. Free Induction Decays were multiplied by an exponential function equivalent to a 0.3 Hz line-broadening factor before applying Fourier’s transformation. The spectra were phased and the baseline was corrected and referenced to the peak of TSP at 0 ppm using TopSpin 4.0.5 (Bruker). One-dimensional spectra ranging from 0.5 to 9.5 ppm were bucketed by intelligent adaptive bucketing and the corresponding spectral areas were integrated using NMRProcFlow (https://www.nmrprocflow.org/). The region between 4.5 and 5 ppm, corresponding to residual water, was removed. Total spectral areas were calculated using the remaining buckets and normalization was carried out by Constant Sum Normalization.

### Nuclear Magnetic Resonance metabolite identification

Metabolites were assigned using Chenomx Profiler NMR 8.4 with reference to the Human Metabolome Database [22–24] and Biological Magnetic Resonance Data Bank [25, 26]. Potential candidate compounds were also validated using standards. If necessary, standard two-dimensional experiments (^1^H-^1^H homonuclear total correlation spectroscopy (TOCSY) and ^1^H-^13^C heteronuclear single quantum coherence sequences (HSQC)) were performed using a Bruker Ascend 700 MHz spectrometer (Massachusetts, USA) coupled to a Bruker AVANCE NEO console equipped with a 5 mm triple resonance probe at 298 K. TOCSY two-dimensional spectra were acquired following a 60 ms spin-lock pulse, 32 scans, 2048 data points, 156 increments with a spectral width of 7796 Hz. HSQC spectra were acquired through 164 scans, 164 data points, 256 increments with a spectral width of 7812 Hz (^1^H, 13 ppm) and 36220 Hz (^13^C, 240 ppm).

### Statistical analysis

For data analysis, reduction, and the determination of relevant metabolites, distinct multivariate and univariate statistical analyses were performed. All variables were mean-centered and divided by their standard deviation prior to multivariate analysis. Principal component analysis (PCA) and partial least squares-discriminate analysis (PLS-DA) were performed to obtain overview changes in metabolomes using MetaboAnalyst 4.0 [27]. Unsupervised PCA was performed on the data matrix to visualize samples in a reduced dimensionality, find clusters, and identify potential outliers. Hierarchical clustering analyses were performed using Ward’s method combined with Euclidean distance measure. Supervised analyses (PLS-DA) were then performed to maximize the covariance between the data matrix and the time group variable. The number of components was determined by cross-validation, which yielded model goodness of fit (R^2^) and predictability (Q^2^). The results of these two multivariate analyses were reported in terms of component scores. In the score plots, each point corresponds to a sample spectrum. Multiblock PCA, combining both NMR and LC-MS datasets, was performed using the MBAnalysis 2.0.2 package in R 4.3.2. Statistical differences between different sample groups were estimated using one-way ANOVA followed by Tukey’s post-hoc test, performed using GraphPad 9. Statistical differences were considered significant when p<0.05.

## Results

### Patient cohort and liver tissue sample cryopreservation intervals

**Fig 1:**
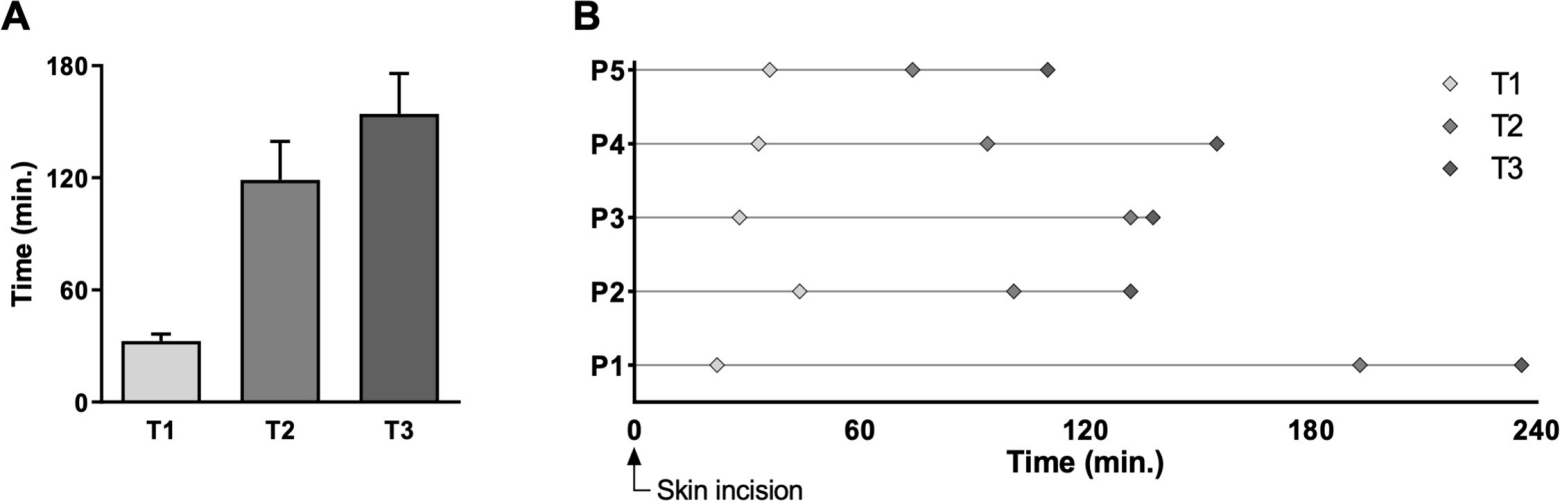
Cryopreservation timepoints and average delay between sample cryopreservation and time at skin incision. Average times for T1 (cryopreservation before liver transection), T2 (cryopreservation after liver resection), and T3 (cryopreservation after liver resection and on-ice delay) (n = 5) (A). Individual cryopreservation timepoints for T1, T2, and T3 samples for each patient (B).

Liver tissue samples were collected from 5 patients, among which were 3 males and 2 females, with a median age of 75 years (mean 73.8 ± 2.7 years). During surgery, samples were cryopreserved at three timepoints relative to the time at skin incision (T0). Samples were cryopreserved at T1, prior to liver transection, at T2, directly after liver resection, and at T3 in the Pathology laboratory after completion of routine macroscopic examination. Average time for sample collection from skin incision was 32.6 ± 3.7 minutes for T1, 118.8 ± 20.8 minutes for T2, and 154.2 ± 21.7 minutes for T3 (Fig 1A). As seen in Fig 1B, significant variability was sometimes observed between samples. For example, T2 for patient 1 was 193 minutes and was 132 minutes for patient 3, which were considerably closer to mean T3 than T1.

### Time at cryopreservation is the principal source of variability in liver tissue metabolomic data

**Fig 2:**
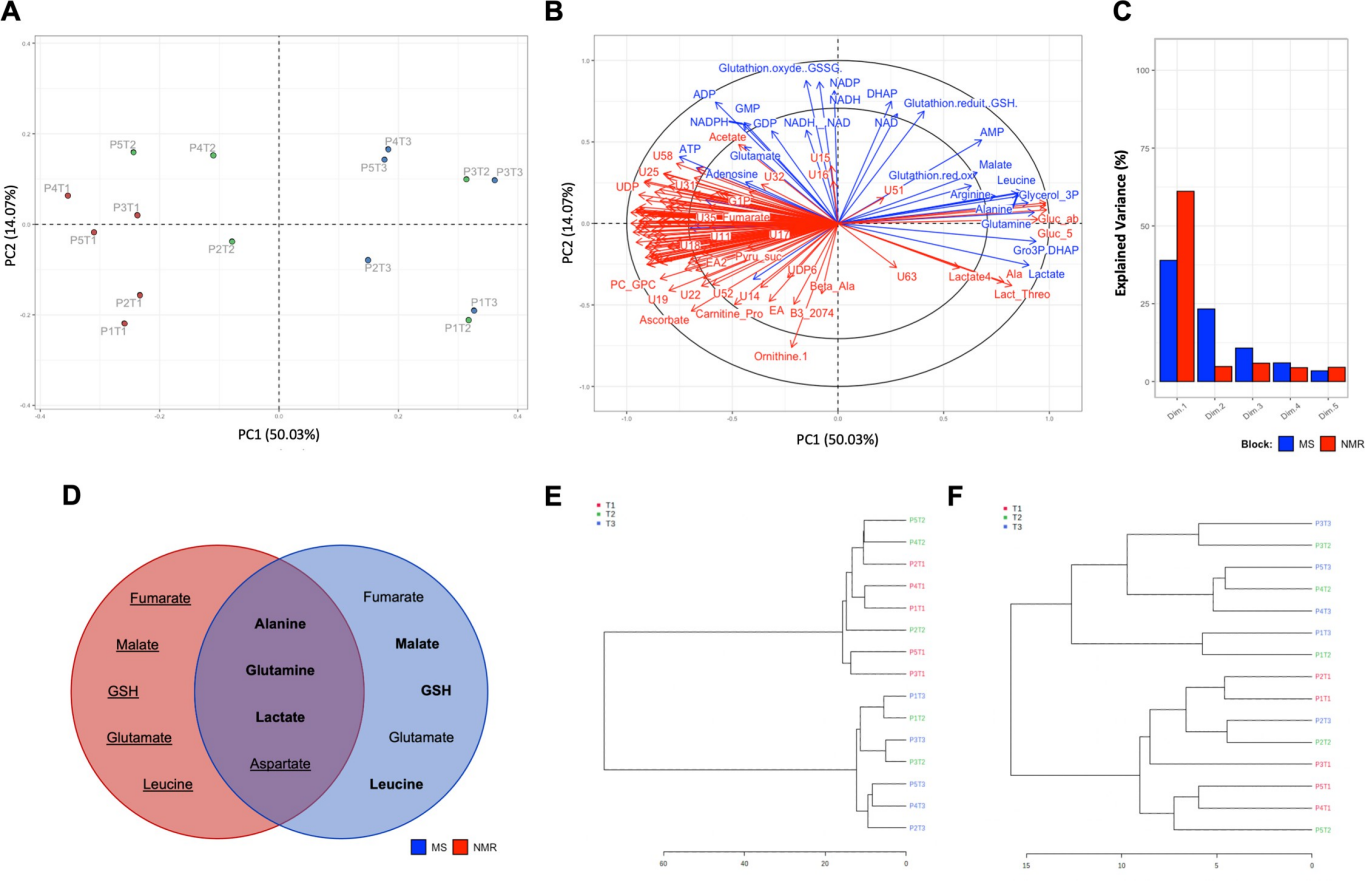
Unsupervised multiblock principal component analysis (PCA) combining NMR and LC-MS metabolomic datasets. Scores plot based on the combined NMR and LC-MS data matrices (A) colored with respective group projections according to the time of sample collection (T1, T2, and T3). Loadings plot depicting the total contribution of detected/identified metabolites in NMR (red) and LC-MS (blue) to sample positioning in the multiblock scores plot (B). Principal components explaining data variability in NMR (red) and LC-MS (blue) independently (C). Venn-diagram of overlapping metabolites detected in NMR (red) and LC-MS (blue) metabolomics, with comparison of increased (bold), decreased (underline) or unchanged metabolites in the T3 group compared to the T1 group (D). Hierarchical clustering of samples are represented as dendrograms for NMR (E) and LC-MS (F) datasets.

First, an unsupervised multi-block principal component analysis (PCA) was performed combining both metabolomic datasets to identify major sources of variability between liver samples (Fig 2A–2C). The data were visualized in a principal component scores plot to identify metabolic trends and possible outliers. The scores plot from the multiblock analysis revealed a principal component 1 of 50.03%, meaning that a single factor was responsible for 50.03% of the variability observed between liver samples (Fig 2A). A second component, PC2, was revealed to contribute to 14.07% of data variability between liver samples when analyzing NMR and LC-MS in combination (Fig 2A). Additional components, namely PC3, PC4, and PC5, explained 8.33%, 5.22%, and 4.01% of metabolomic variability, respectively. The identification of metabolite profiles of liver samples according to their respective times of cryopreservation revealed a segregation of groups along the PC1 axis, revealing that time was the variable responsible for most of the variability in our metabolomic data. In fact, T1 (red) and T3 samples (blue) were distinctively clustered, whereas T2 samples (green) overlapped with samples from both T1 and T3 groups (Fig 2A). Clustering of patients’ samples according to the time at cryopreservation was especially obvious for the T1 group. In Fig 2B, the multiblock PCA loadings plot identifies metabolites that were consistently associated with liver samples, either identified from the NMR (red) or LC-MS (blue) datasets. In fact, both analyses were complementary in terms of detected or identified metabolites, though certain features did overlap. Multiblock PCA also revealed principal components attributable to data variability in an independent fashion, namely either for the NMR dataset or the LC-MS dataset (Fig 2C). For NMR metabolomics, 80.85% of sample variability was attributable to the combined first 5 components: 61.12% (1^st^ dimension), 4.82% (2^nd^ dimension), 5.89% (3^rd^ dimension), 4.45% (4^th^ dimension), and 4.57% (5^th^ dimension). For the LC-MS dataset, the first 5 components represented a combined 81.90% of sample variability: 38.4% (1^st^ dimension), 23.31% (2^nd^ dimension), 10.76% (3^rd^ dimension), 5.99% (4^th^ dimension), and 3.44% (5^th^ dimension). In Fig 2D, certain metabolites having been identified in both NMR and LC-MS modalities showed redundant patterns over cryopreservation delay, as seen in Fig 2B. Overlapping metabolites, such as lactate and alanine which tended to increase over time as identified through both analyses, are represented within a Venn diagram (Fig 2D). Additionally, hierarchical clustering analysis was performed for the studied samples to identify if distinctive clustering occurred between sample times, as depicted in dendrograms for NMR (Fig 2C) and LC-MS (Fig 2F) datasets. Briefly, T1 and T3 samples were separated into two distinct classes for NMR spectroscopy, with T2 samples being classified as either belonging to the T1 or T2 clusters. The T2 samples for patients 1 and 3 were clustered with their respective T3 samples.

### NMR-based metabolomics reveal a global metabolomic signature of hepatic tissue adaptation over time after sampling

**Fig 3:**
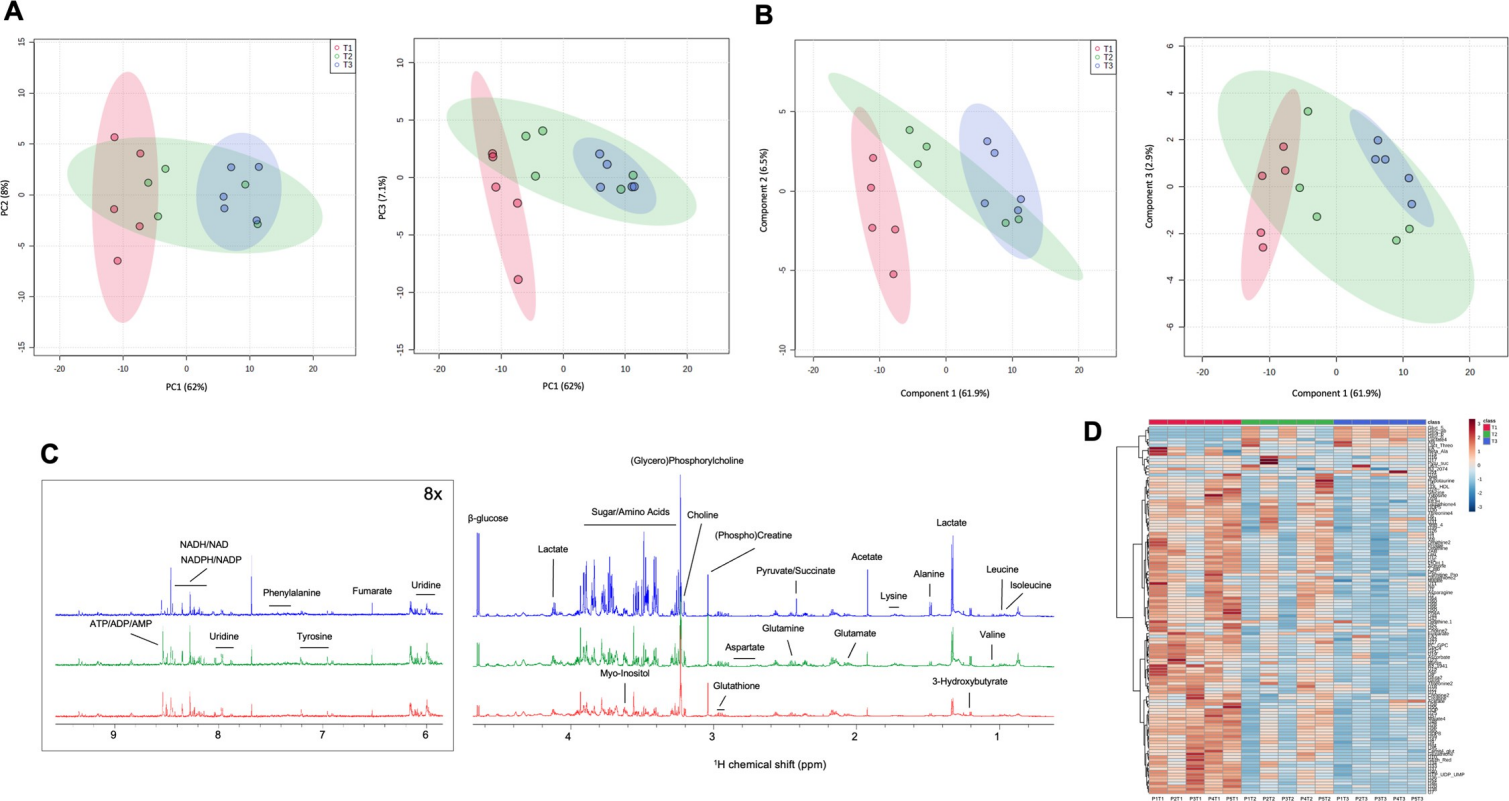
Representative 700 MHz ^1^H NMR spectra of tissue samples. Scores plots obtained from unsupervised principal component (A) and supervised partial least squares-discriminant analysis (PLS-DA, B) based on NMR data matrix; R^2^ = 0.73, Q^2^ = 0.60. Representative 700 MHz ^1^H NMR spectra of tissue samples belonging to patient 5 (δ1-δ4.5 and 7x-magnification of δ6-δ9 extension) are shown in red at T1, green at T2 and blue at T3 (C). Heatmap representation of the relative abundance of 130 spectral features as extracted by PLS-DA datasets (D).

To establish an unbiased metabolic profile of samples, we used a high-throughput NMR profiling approach. This allowed for the identification of a global signature of liver sample metabolites over time until cryopreservation. In support of the multiblock PCA analysis combining both metabolomics approaches, PCA was performed on the NMR dataset independently of the LC-MS dataset (Fig 3A). Samples from the T1 and T3 timepoints and their respective 95% confidence interval regions were distinctly separated along the PC1 axis, which represented 62% of data variability. The 95% confidence interval region of the T2 group overlapped with those of the T1 and T3 groups. Additional components, PC2 (8%) and PC3 (7.1%), contributed less than the PC1 to data variability (Fig 3A). Conversely, the identification of metabolite profiles of liver tissue samples according to individual patients rather than time showed no distinct clustering along the PC1 (S2 Fig).

A projection to latent structures model was performed using partial least squares-discriminant analysis (PLS-DA) to identify significant variations in liver metabolic profiles between each time point along tissue sampling. In this model, depicted in Fig 3A, all spectral features were included as the descriptor variables, and the time of sample collection was used as the response variable. Model reliability was adequate as assessed using explained variance (R^2^ = 0.73) and predictive capability (Q^2^ = 0.60) (Fig 3B).

We first obtained NMR spectra of aqueous liver extracts at the three timepoints for each patient (Fig 3C and S1 Fig). The spectra were dominated by typical metabolites found in liver extract samples such as amino acids, organic acids and bases, as well as carbohydrates (Fig 3C). As seen in Fig 3B, certain metabolites exhibited an apparent change over time during the sample collection. Importantly, lactate and β-glucose accumulated in liver tissue over time. Metabolites such as pyruvate, succinate, acetate, and the amino acid alanine showed a similar trend. Conversely, uridine and myo-inositol were among the metabolites that tended to decrease due to cryopreservation delays.

To provide a global overview of metabolite levels in liver samples, a heatmap representation was used to depict the area under the curve of 130 spectral features, representing the relative abundance of a diverse set of metabolites (Fig 3D). Metabolite levels are shown on each row whereas each column corresponds to a single sample of patients (P1-5) and time points (T1-3). Among 130 metabolomic features, a global reduction in the levels of most intracellular metabolites occurred over time, such as UTP/UDP/UMP, ornithine, carnitine, asparagine, and threonine. Glucose and lactate were among the few metabolites that tended to accumulate in liver samples over the same period (Fig 3D). Moreover, T2 samples for patients 1 and 3 exhibited a more similar metabolite profile to all T3 samples rather than the T2 samples obtained from the 3 other patients. Altogether, NMR-guided metabolite profiling revealed that cryopreservation delays considerably influence liver sample metabolomics, as seen by alterations of a broad ensemble of metabolites.

### LC-MS metabolite profiling identifies precise and significant changes in intracellular metabolite levels over time required for liver tissue sampling

**Fig 4:**
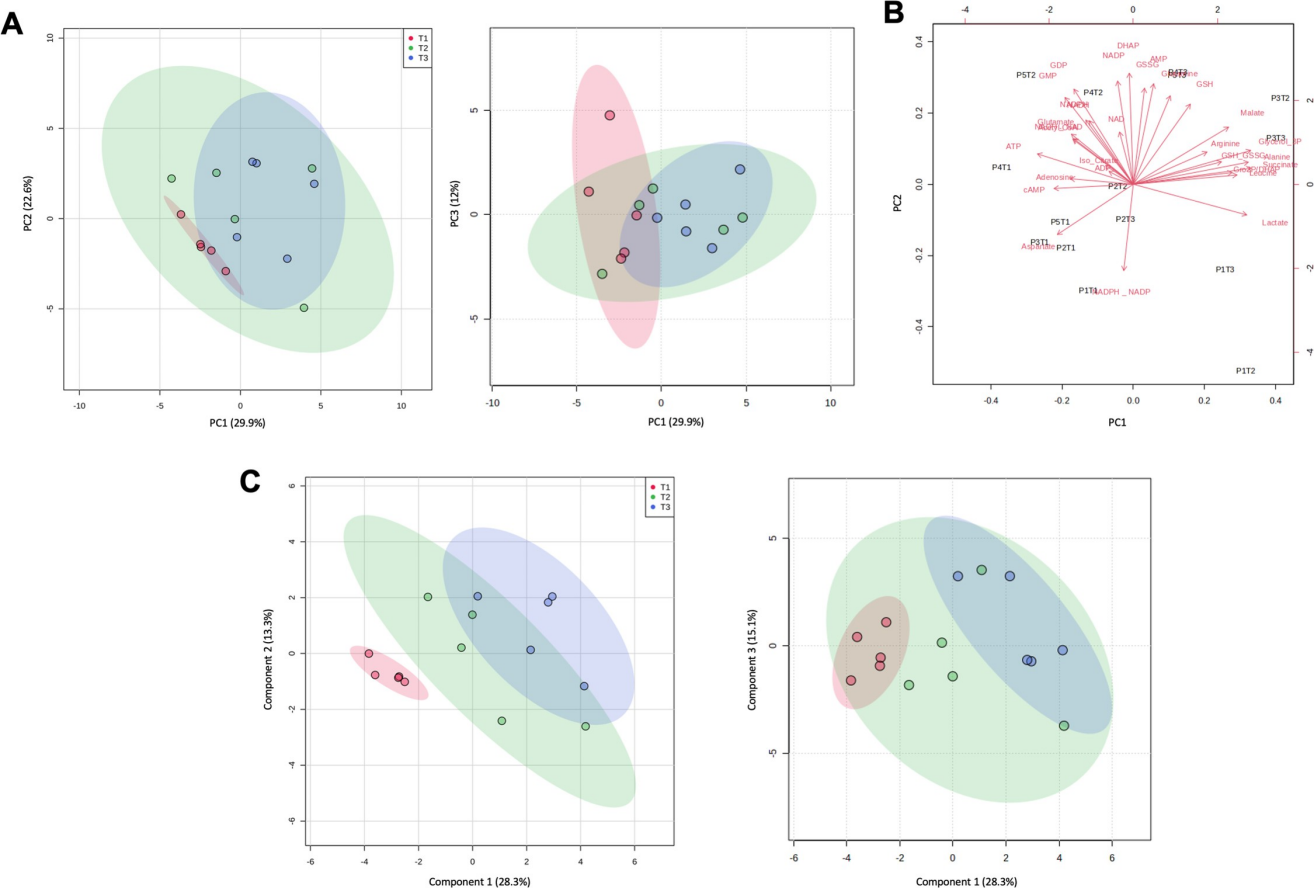
Targeted LC-MS metabolite profiling. Scores plots (A) and loadings plot (B) obtained from unsupervised principal component analysis based on LC-MS data matrix. Scores plots of supervised partial least squares-discriminant analysis of LC-MS metabolomes (C); R^2^ = 0.99, Q^2^ = 0.55.

To precisely characterize changes in metabolite levels over time using ^1^H NMR untargeted profiling, we analyzed all liver extract samples using an LC-MS targeted approach. A total of 30 metabolites were quantified, including amino acids, TCA cycle intermediates, as well as energy-related metabolites. As performed on the NMR dataset, PCA analysis was performed for LC-MS metabolomes independently of the multiblock approach (Fig 4A). Sample clustering was less obvious than in both multiblock PCA and NMR PCA, though T1 and T3 groups did cluster along the PC1 axis (29.9%); the PC2 axis represented 22.6% of data variability whereas PC3 explained that of 12%. In Fig 4B, the contribution of all quantified metabolites to the separation of samples according to their metabolomic profile is represented on a loading plot, mainly revealing metabolite abundance patterns between the T1 and T3 groups. Metabolites that contributed most to the metabolomic discrimination of tissue samples are farther away from the origin (Fig 4B). For example, abundant tissue glutamate, aspartate, and ATP tended toward T1 sample identification, whereas malate, lactate, alanine, and succinate tended toward T3 samples (Fig 4B). Similarly to that observed through NMR metabolomics, inter-individual variability showed no distinct clustering along the PC1 (S2 Fig). Then, supervised classification of LC-MS metabolomes using a PLS-DA model was performed, as shown in Fig 4C. Though T1 and T3 samples were distinctively clustered along the principal component 1, parameters identifying model performance (goodness of fit (R^2^) = 0.99 and predictability (Q^2^) = 0.55) were inconsistent and the PLS-DA model was deemed to be overfitted for the LC-MS data matrix (Fig 4C).

**Fig 5:**
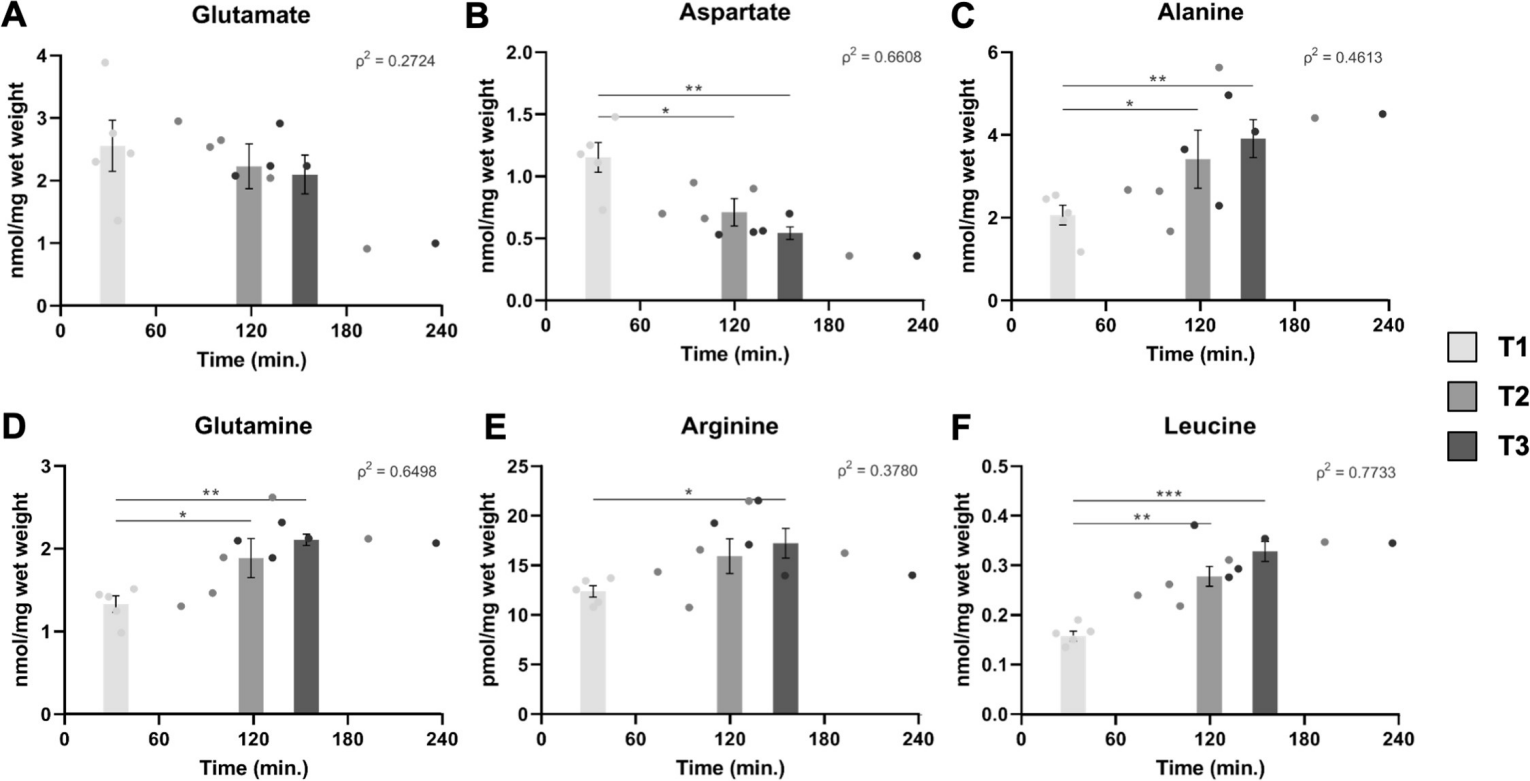
Identification of variations in intrahepatic amino acid levels over time. Quantification of total amino acids glutamate (A), aspartate (B), alanine (C), glutamine (D), arginine (E), and leucine (F) by LC-MS. *: p<0.05; **: p<0.01; ***: p<0.001.

**Fig 6:**
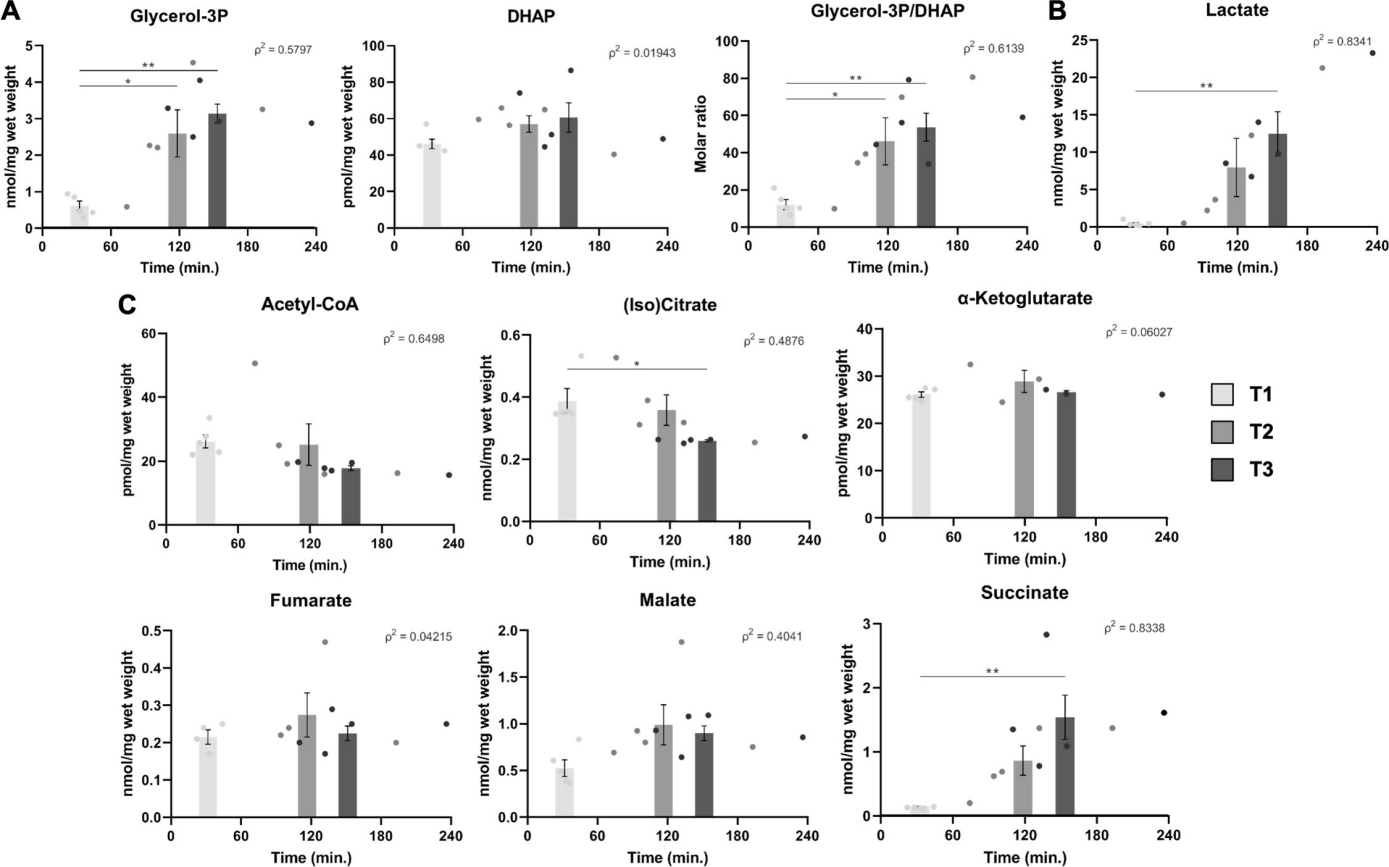
Quantification of glycolysis and TCA cycle intermediates in sampled liver tissue during cryopreservation delay. LC-MS-based quantification of intrahepatic levels of glycerol-3-phosphate (Gro3P), dihydroxyacetone phosphate (DHAP), Gro3P/DHAP ratio (A), lactate (B), acetyl-CoA, (iso)citrate, α-ketoglutarate, fumarate, malate, and succinate (C). *: p<0.05; **: p<0.01; ***: p<0.001.

**Fig 7:**
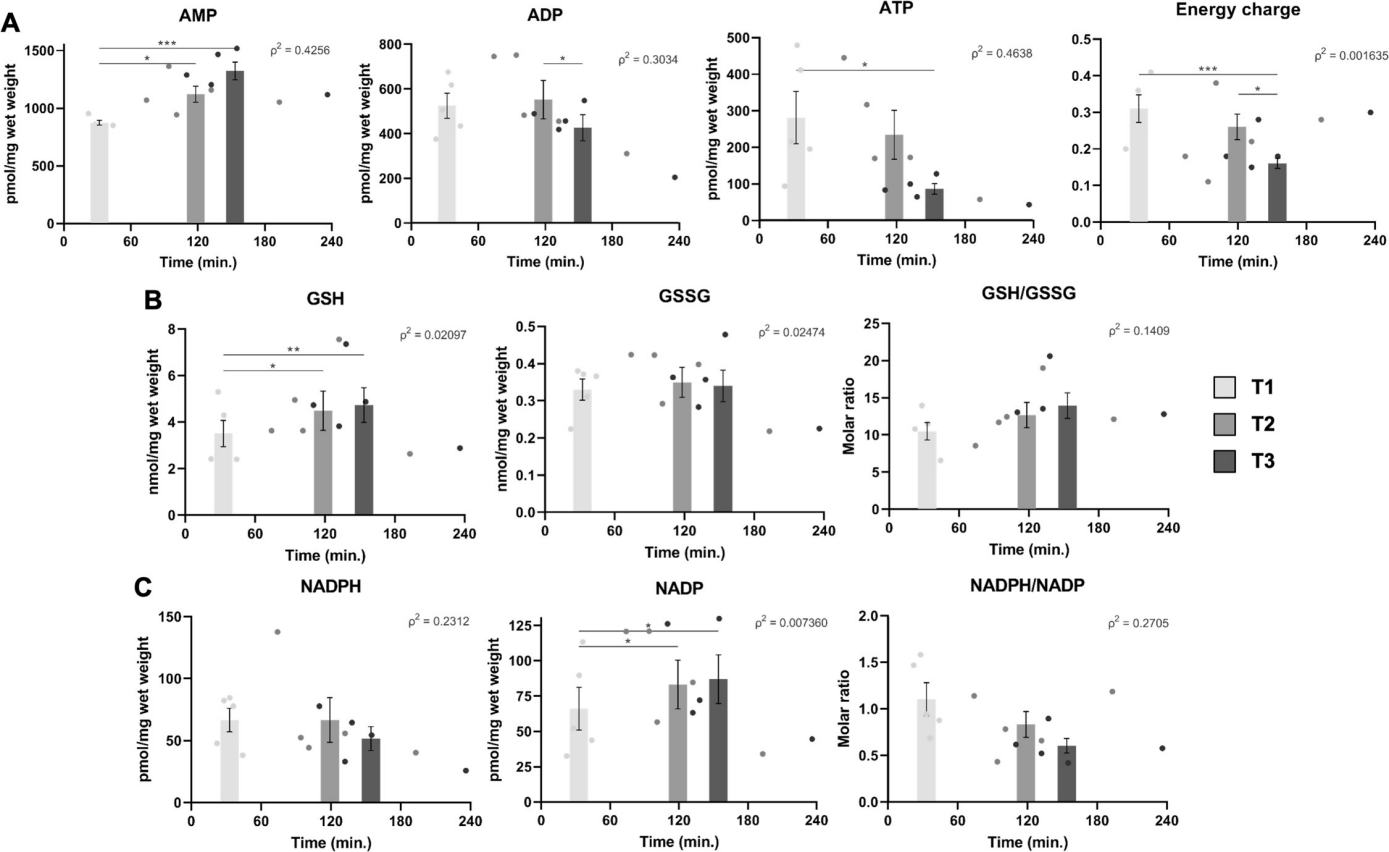
Metabolomics quantification of intrahepatic energy and redox metabolites over time during liver tissue biobanking. Quantification of total AMP, ADP, ATP, energy charge (A), reduced glutathione (GSH), oxidized GSH (GSSG), GSH/GSSG ratio (B), NADPH, NADP, NADPH/NADP ratio (C) by LC-MS. Energy charge of liver samples was calculated as follows: ([ATP] + ½[ADP]) / ([ATP] + [ADP] + [AMP]). *: p<0.05; **: p<0.01; ***: p<0.001.

To identify which metabolites explained metabolomic variance between sample groups, univariate analyses were performed on the LC-MS dataset, given the PLS-DA predictive model was not robust. Indeed, quantitative LC-MS metabolomics revealed a significant change in the level of several intracellular metabolites over time, independently of their contribution to the model for discrimination of liver samples over time during cryopreservation delay (Figs 5–7 and S2 Fig). First, 6 amino acids were quantified in samples. Intrahepatic glutamate tended to decrease during cryopreservation delay, albeit not reaching statistical significance (Fig 5A), whereas aspartate greatly decreased over time (Fig 5B; T1 v. T2, p<0.05; T1 v. T3, p<0.01). As seen in Fig 5C through 5E, there was a time-dependent accumulation of the non-essential amino acids alanine (T1 v. T2, p<0.05; T1 v. T3, p<0.01), glutamine (T1 v. T2, p<0.05; T1 v. T3, p<0.01), and arginine (T1 v. T3, p<0.05). Likewise, the essential amino acid leucine significantly increased during cryopreservation delay (Fig 5F; T1 v. T2, p<0.01; T1 v. T3, p<0.001).

Next, the levels of glycolysis pathway and glucose-derived metabolites were measured. The concentration of Gro3P was significantly higher in liver samples collected after liver resection (T2) and pathology exam (T3) compared to those collected before liver transection (T1) (Fig 6A; T1 v. T2 and T3, p<0.01), whereas dihydroxyacetone phosphate levels were only slightly higher with time (Fig 6A). As such, the Gro3P/DHAP ratio significantly increased over time (Fig 6A; T1 v. T2, p<0.05; T1 v. T3, p<0.01). Also, as seen in Fig 6B, intrahepatic lactate increased in a time-dependent fashion from 0.47 ± 0.15 at T1, to 7.99 ± 3.89 at T2, to 12.48 ± 2.96 nmol/mg wet weight at T3 (T1 v. T3, p<0.01).

Furthermore, quantitative analysis of six among the nine main TCA cycle intermediates was also performed. Among these, levels of acetyl-CoA declined at T2 and T3 compared to T1 (Fig 6C; p>0.05). Those of (iso)citrate also significantly decreased over time (Fig 6C; T1 v. T3, p<0.05), whereas intermediates α-ketoglutarate, fumarate, and malate remained stable. Interestingly, succinate, the product of energy-yielding succinyl-CoA synthetase, increased from 0.13 ± 0.0041 at T1, to 0.85 ± 0.23 at T2, to 1.53 ± 0.35 nmol/mg wet tissue at T3 (Fig 6C; T1 v. T3, p<0.01).

Energy metabolites ATP, ADP, and AMP exhibited apparent and significant variations over cryopreservation times as depicted in Fig 7A, whereas GTP, GDP, and GMP did not (S2A Fig). Indeed, levels of AMP were significantly higher at T2 and T3 (T1 v. T2, p<0.05; T1 v. T3, p<0.001), though ADP levels reduced over time (T2 v. T3, p<0.05). Intrahepatic ATP was decreased from 279.2 ± 71.8 at T1, to 232.4 ± 67.2 at T2, to 83.9 ± 14.4 pmol/mg wet weight at T3 (T1 v. T3, p<0.05). Accordingly, the calculated energy charge of liver samples similarly decreased over T1, at 0.31 ± 0.038, through T2, at 0.26 ± 0.035, and T3 with an energy charge of 0.16 ± 0.014 (Fig 7A; T1 v. T3, p<0.001; T2 v. T3, p<0.05). NADH and its oxidized form NAD were stable over the three timepoints, such that the NADH/NAD ratio was not significantly altered (S2D–S2F Fig).

Glutathione, a major substrate in redox homeostasis and detoxication, was also altered due to delays in cryopreservation of liver tissue samples. Namely, its reduced and active form (GSH), significantly increased over time (Fig 7B; T1 v. T2, p<0.05; T1 v. T3, p<0.01). The oxidized form of glutathione (GSSG) and the resulting GSH/GSSG ratio, however, did not undergo significant changes (Fig 7B). As GSSG recycling to reduced GSH requires NADPH as a cofactor, we measured reduced NADPH and oxidized NADP in our biospecimens. As seen in Fig 7C, whereas NADPH exhibited a decreasing trend over time, NADP significantly increased (T1 v. T2 and T3, p<0.05), such that the NADPH/NADP ratio showed an apparent decrease during the same period, although not reaching statistical significance. Lastly, the absolute abundance of adenosine and cAMP were slightly lower at T2 and T3 compared to T1, whereas malonyl-CoA remained unchanged (S2C–S2E Fig).

Overall, targeted LC-MS metabolomics of liver tissue samples confirmed, with untargeted NMR-based metabolomics, that time at cryopreservation after tissue collection during liver sampling has a significant impact on the metabolic makeup of liver tissue.

## Discussion

Flexibility and adaptability are among the virtues of liver metabolism. Given the vital role of the liver in regulating systemic metabolism, hepatocytes adapt to an array of physiological states and environmental constraints. Interestingly, the hypothesis that liver metabolism alterations occur during the course of disease has raised considerable interest in hepatology research. To grasp the clinical significance of such alterations, among other characteristics of liver pathobiology, researchers have been exponentially dependent on the availability of established biobanks that offer a library of biological samples and clinical data fundamental to studying liver pathogenesis and identifying novel disease biomarkers. Nonetheless, standardized procedures for liver tissue sampling and subsequent biobanking are lacking. Indeed, preanalytical processes and sample storage conditions can have deleterious effects on the reproducibility and reliability of data acquired from biobank specimens [13, 15, 28, 29]. For example, freeze/thaw cycles and tissue sample stability are critical to maintain cell, protein, and metabolic integrity of collected specimens [28].

In this study, we characterized the metabolic profiles of liver tissue samples collected from patients over three timepoints during liver resection surgery to assess the impact of cryopreservation delays on liver tissue metabolomics. Interestingly, complementary metabolomic profiling analyses through NMR and LC-MS showed alterations of the liver metabolome over time as well as a clustering of samples based on the delay between tissue sampling and cryopreservation. This highlights that delays in cryopreservation represented the most important variable for metabolomic alterations occurring during liver biobanking, which supersedes interindividual variations. Multiblock PCA, combining both NMR-based and LC-MC-based metabolomic profiling strategies, identified two samples included in the T2 group within the T3 projection, meaning that their metabolomic profiles were more similar to those of the T3 group than the other T2 samples. In fact, these samples belonged to patients 1 and 3, who, as seen in Fig 1B, had considerable delays compared to the T2 timepoints of the three other patients. Indeed, timepoints of both outlier T2 samples were much closer to T3 than T1, which further underlines the influence of time-dependent extracorporeal alterations of liver metabolism on the hepatic metabolome. Indeed, this suggests that timing delays contribute the most to metabolic alterations of liver specimens during biobanking protocols, regardless of if cryopreservation is performed before or after pathology analysis. With regards to sample groups, biospecimens frozen after analysis in pathology had considerably altered metabolomes compared to those cryopreserved immediately after resection, and even more-so when compared to those collected prior to liver transection. These findings highlight the importance of considering delays that occur prior to liver sample cryopreservation during tissue biobanking, as metabolic alterations of hepatic tissue occur rapidly and significantly both during surgery and after surgical removal.

To obtain a more detailed picture of these metabolic alterations, NMR spectroscopic datasets were condensed into a heatmap depicting the abundance of all spectral features (Fig 3D and S1E Fig). Such untargeted metabolomics showed that there was an important modification of global metabolic tissue signatures from T1 through T2 and T3. Very few metabolites tended to increase over time, whereas the abundance of most metabolites gradually decreased over cryopreservation times T2 and T3 compared to T1. These findings suggest that endogenous adaptations of liver metabolism during surgery and cryopreservation delay induce alterations in a broad set of metabolites, which could, at least partially, be a result of tissue ischemia. A previous study investigating the role of ischemia on the NMR metabolome of liver tissues reported significant modifications of 16 metabolites, including amino acids, glucose, and GSH [30]. Numerous cellular pathways have also been shown to be altered under ischemic conditions, which in turn influence the metabolic composition and activity of tissues [12, 31–34]. In the liver, ischemia has been shown to induce anaerobic glycolysis, impaired mitochondrial respiration, inflammation-promoting perturbations of lipid metabolism, as well as alterations in the tryptophan-metabolizing kynurenine pathway [35, 36]. Ischemia broadly represses oxidative metabolism of various intermediates, including fatty acids, carbohydrates, and amino acids [37]. Cell death, which may occur as a consequence to loss of perfusion and anoxia after specimen excision, is also associated with specific metabolic hallmarks [38]. For example, mitochondrial dysfunction, which may arise from ischemia, is linked with the induction of apoptosis [39]. Additionally, drastic metabolic alterations occur during primary hepatocyte isolation, such that the latter express significantly different metabolic activities compared to those of hepatocytes *in situ* [40]. Accordingly, the metabolomic signature of *in situ* liver tissue would be lost when important delays occur between sample collection and liquid nitrogen storage.

As NMR analysis identified a metabolic pattern associated with liver sample cryopreservation delay, we proceeded to dissect the time-dependent alteration of liver tissue metabolomic profiles into individual metabolites. LC-MS-based analyses quantified 30 intermediates belonging to diverse metabolic pathways. Similarly to NMR profiling, unsupervised analysis of LC-MS data, through PCA, showed distinct T1 and T3 sample clustering along the first component. Supervised analysis of LC-MS data showed an overfitted PLS-DA model as suggested by a considerable inconsistency between goodness of fit and predictability, which could be explained by the limited number of samples, but mostly due to the limited quantified metabolites in LC-MS as the NMR PLS-DA was indeed robust. Consequently, to identify significant metabolites through LC-MS profiling, we performed univariate analyses rather than identifying important metabolites in the PLS-DA model given its lacked predictive performance.

We first identified specific changes of amino acid levels in liver tissues during cryopreservation delays. Glutamine increased over time whereas glutamate tended to decrease, suggesting an arrest of glutaminolysis, the breakdown of glutamine into glutamate. In periportal hepatocytes, glutaminolysis releases ammonia which is then incorporated into the urea cycle through carbamoyl phosphate synthetase 1 [41, 42]. Also, accumulation of arginine over time could be explained by decreased urea cycle activity within hepatocytes during delays in cryopreservation of liver tissue. Similarly, alanine and leucine significantly increased over time, alanine also being identified as being among the prominent metabolites in T2 and T3 samples in NMR. The accumulation of such amino acids could signify a loss of protein synthesis, which is one of the fundamental functions of normal liver. Increased alanine may also be a result of a decrease in pyruvate demand, hypoxia contributing to the shunting of mitochondrial metabolism and the TCA cycle. Aspartate was the single studied amino acid that decreased over time. Consistent with this observation, previous studies have reported aspartate loss as being a hallmark of hypoxia and liver ischemia [30, 43].

Glycolysis and TCA cycle were also subject to major disturbances during specimen cryopreservation delay. Interestingly, NMR metabolite profiling identified β-glucose as accumulating in liver samples during storage delays. This could arise from glycogen storage breakdown as a compensatory mechanism to loss of tissue perfusion. In fact, accumulation of glucose and glycolytic intermediates have recently been reported as a prominent feature of brain ischemia/reperfusion-induced metabolic disturbance as well as during liver ischemia [30, 44]. A decreasing trend in acetyl-CoA, the product of the pyruvate dehydrogenase complex and an important substrate for citrate synthesis, suggests that TCA cycle anaplerosis from glucose was disturbed over time. Instead, glucose flux was redirected toward lactate fermentation during cryopreservation delay, suggesting that mitochondrial metabolism is replaced by an increased dependence on anaerobic glycolysis. Lactate accumulation through anaerobic glycolysis may also contribute to decreasing intracellular pH, which has been shown to limit cell death during liver ischemia [39]. Furthermore, within the Krebs cycle, succinate significantly accumulated over time in both NMR and LC-MS spectroscopic datasets. Interestingly, accumulation of succinate has been described as a hallmark of ischemia in the liver among other organs [32, 35].

In line with TCA cycle disruption, energy metabolites were altered during cryopreservation delay. Whereas AMP significantly increased over time, both ADP and ATP metabolites decreased; the calculated energy charge was decreased at T2, which was more pronounced at T3. This suggests that continuous cellular metabolic activity of hepatocytes before cryopreservation depletes ATP, which in turn may halt a plethora of metabolic pathways. Decreased ATP implies impaired activity of oxidative phosphorylation, which could be a result of ischemia. Indeed, damage and dysfunction of mitochondrial complexes have been shown to appear rapidly during ischemic episodes of the liver [39]. Limiting ADP and ATP levels in liver tissue have also been described as a hallmark of apoptosis during hepatic ischemia [39].

Lastly, metabolites involved in redox homeostasis were altered in sampled liver tissues during cryopreservation delay. Though we observed increased GSH synthesis, as GSH increased and GSSG remained stable, NADP significantly increased over time, suggesting an imbalance in GSH recycling. With regards to the quality of biobank specimens, uncontrolled accumulation of reactive oxygen species could damage biomolecules such as DNA, proteins, and metabolic intermediates. Altered redox homeostasis may occur due to mitochondrial dysfunction during delays between sample excision and cryopreservation, possibly due in part to tissue ischemia [39].

Altogether, cryopreservation delays occurring during biobanking protocols are detrimental to the metabolic composition and activity of liver tissue. In our study, the variability in metabolomes due to delays before liquid nitrogen freezing superseded inter-individual variability, highlighting the importance of referring to standardized sample collection procedures for establishing high-quality liver tissue biobanks. Specifically, accumulation of succinate, lactate, glutamine, arginine, leucine, and alanine as well as decreased citrate, aspartate, and ATP were the most notable hallmarks of cryopreservation delays. Consequently, the key disrupted pathways during cryopreservation delays of liver biobank specimens were oxidative phosphorylation, TCA cycle activity and anaplerosis, glycolysis, urea cycle, glutaminolysis, and protein synthesis. In light of such findings, standardized pre-analytical procedures for human liver tissue biobanking are of paramount importance to obtain reliable metabolomic data that veritably depict *in situ* metabolism. Our metabolomic analyses through NMR and LC-MS have shown that cryopreservation delays may introduce bias to the detection of various metabolites potentially implicated in metabolic reprogramming of liver disease, and as such, global efforts aiming to standardize and record liver biobanking procedures as well as educate biobank specialists on the risks of cryopreservation delays is of significant importance.

## Supporting information

S1 Fig 700 MHz ^1^H NMR spectra of hepatic tissue samples over time during liver biobanking. 700 MHz ^1^H NMR spectra of tissue samples collected at T1 (red), T2 (green), and T3 (blue) throughout δ1-δ4.5 and 7x-magnification of δ6-δ9 extension, for patients 1 (A), 2 (B), 3 (C), and 4 (D).
(PDF)

S2 Fig Relative quantification of ^1^H NMR spectral features of hepatic tissue samples. Heatmap representation of the relative abundance of 130 spectral features extracted from ^1^H NMR datasets.
(TIFF)

S3 Fig Principal component analyses samples annoted as individual patients. Scores plots of ^1^H NMR (A) and LC-MS (B) spectroscopic datasets per principal component analysis, samples identified as belonging to P1 (red), P2 (green), P3 (blue), P4 (cyan), or P5 (magenta).
(TIFF)

S4 Fig Quantification of various intrahepatic metabolites during cryopreservation delay. GMP, GDP, GTP (A), NAD, NADH, NADH/NAD ratio (B), adenosine (C), cyclic AMP (cAMP (D)), and malonyl-CoA (E) were quantified in collected liver samples using LC-MS targeted metabolomics analyses.
(TIFF)
