# Supplementary figures and images for: Impact of the delay in cryopreservation timing during biobanking procedures on human liver tissue metabolomics
Source: PLoS One. 2024 Jun 10;19(6):e0304405. doi: 10.1371/journal.pone.0304405 (PMC11164386; doi:10.1371/journal.pone.0304405)

**A**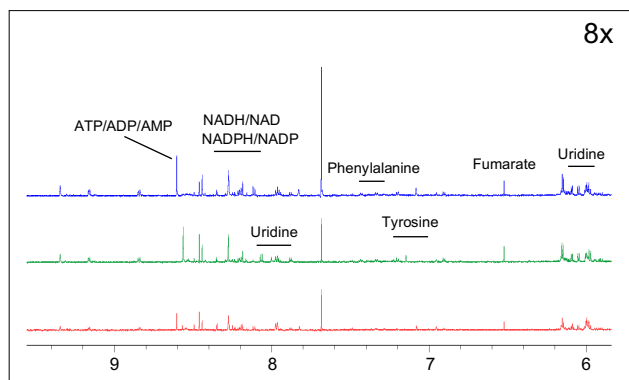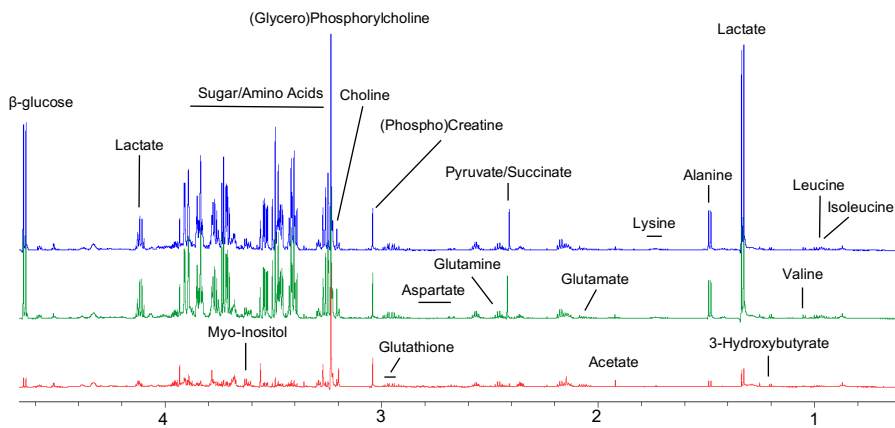**B**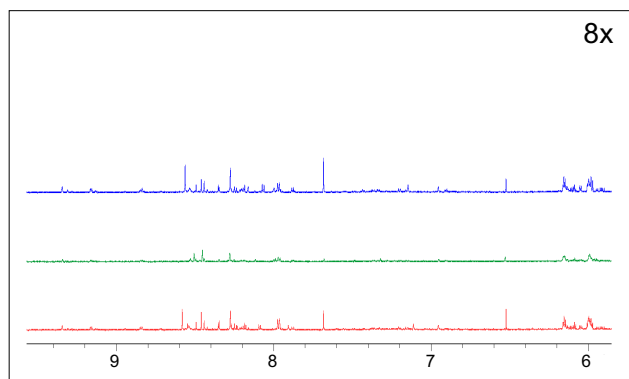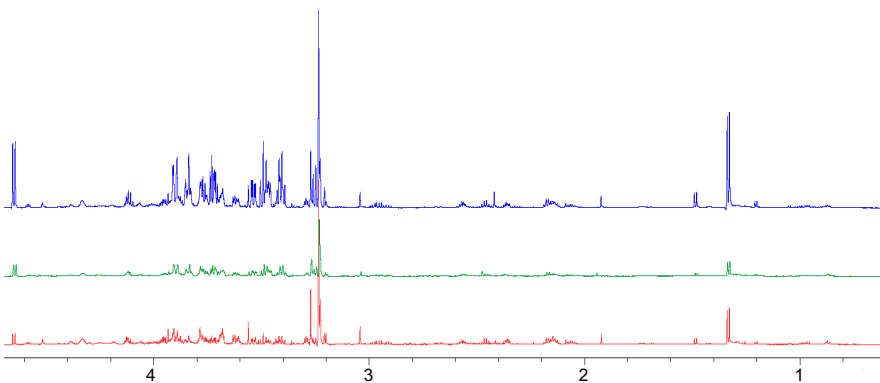**C**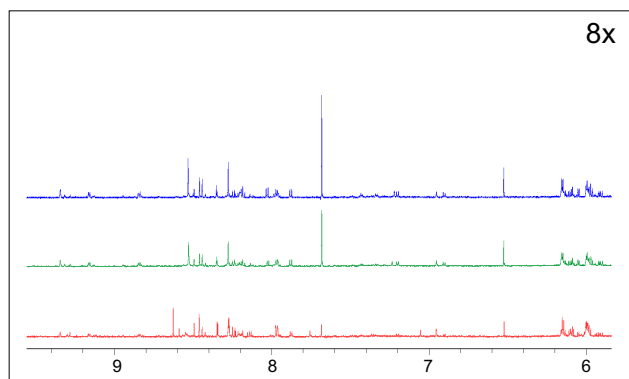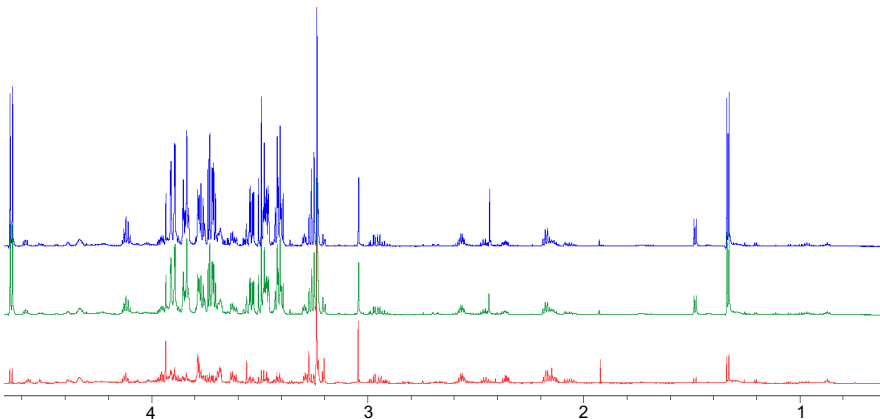**D**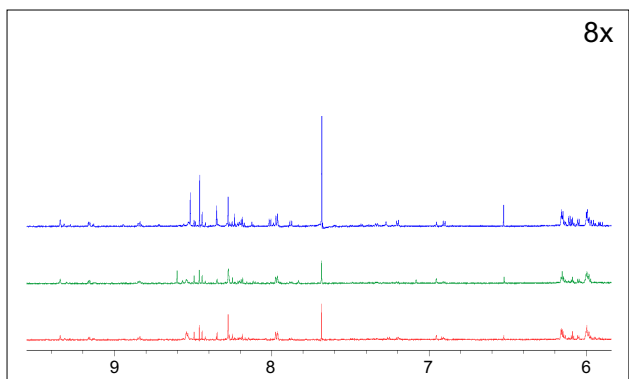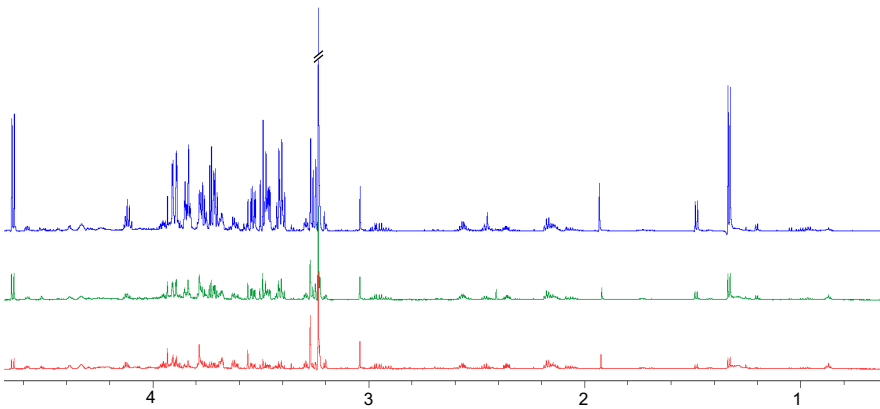

<sup>1</sup>H chemical shift (ppm)

Supplement: S1 Fig — 700 MHz 1H NMR spectra of tissue samples collected at T1 (red), T2 (green), and T3 (blue) throughout δ1-δ4.5 and 7x-magnification of δ6-δ9 extension, for patients 1 (A), 2 (B), 3 (C), and 4 (D). (PDF) [file pone.0304405.s001.pdf]

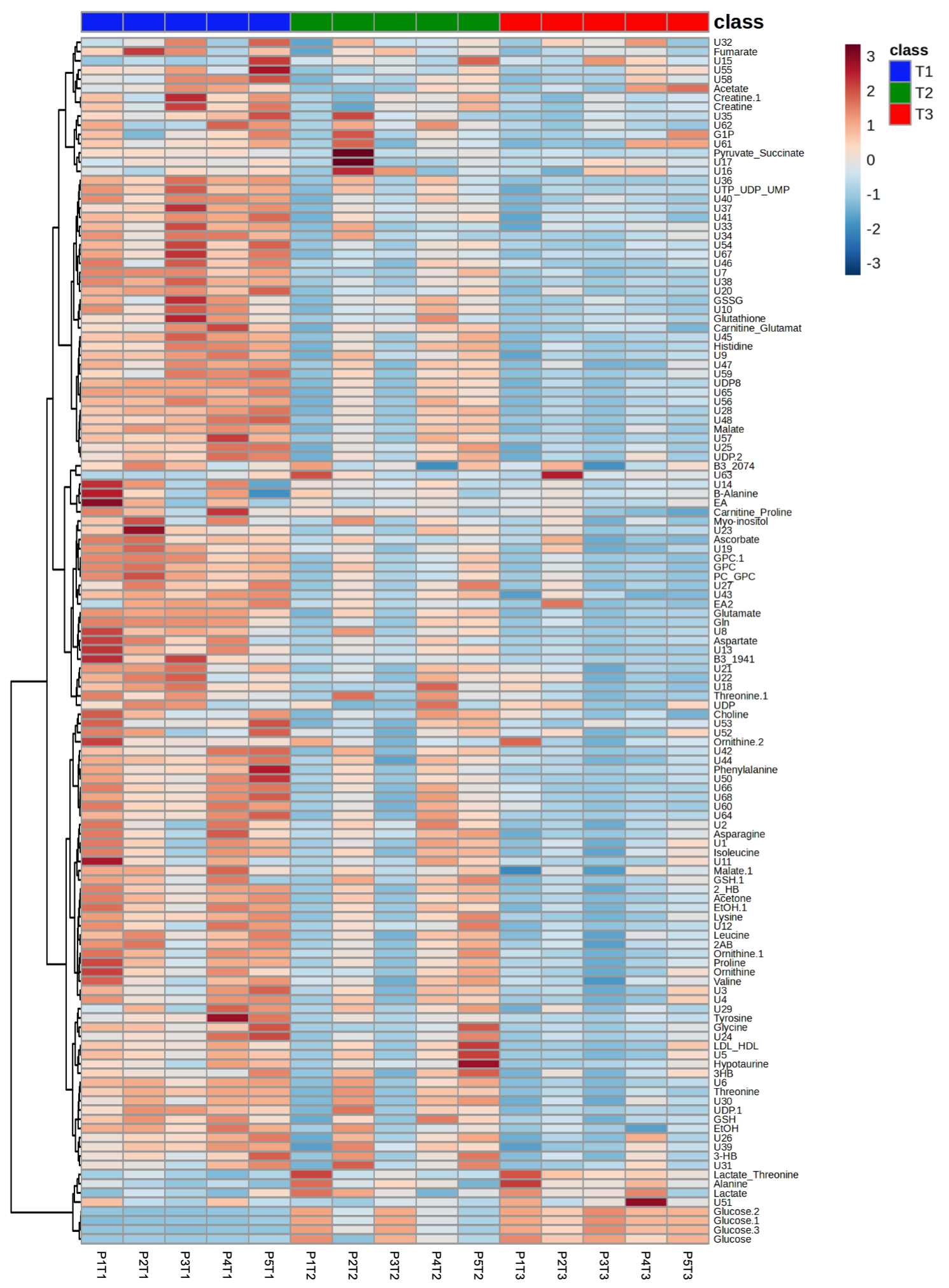

Supplement: S2 Fig — Heatmap representation of the relative abundance of 130 spectral features extracted from 1H NMR datasets. (TIFF) [file pone.0304405.s002.tiff]

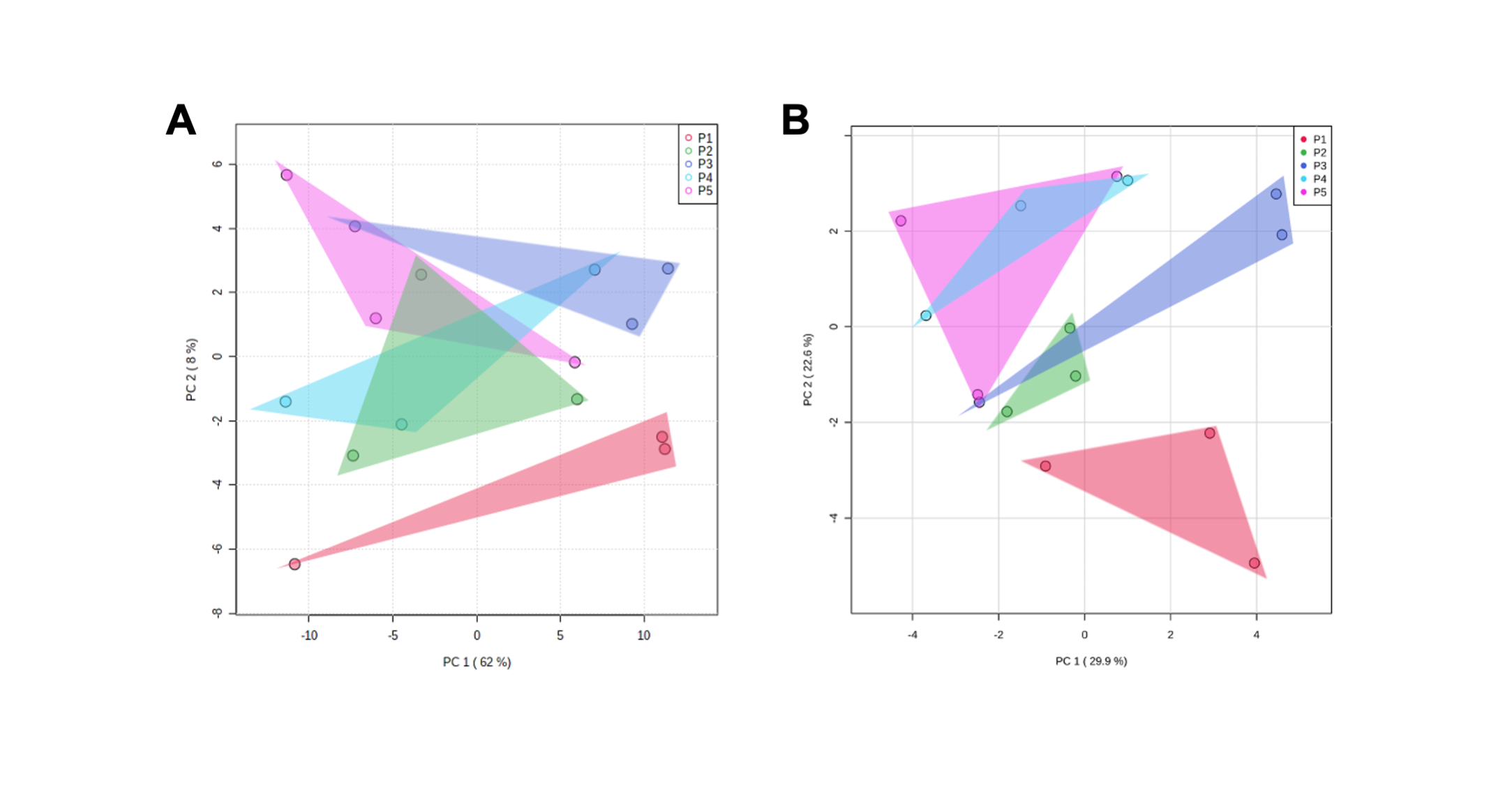

Supplement: S3 Fig — Scores plots of 1H NMR (A) and LC-MS (B) spectroscopic datasets per principal component analysis, samples identified as belonging to P1 (red), P2 (green), P3 (blue), P4 (cyan), or P5 (magenta). (TIFF) [file pone.0304405.s003.tiff]

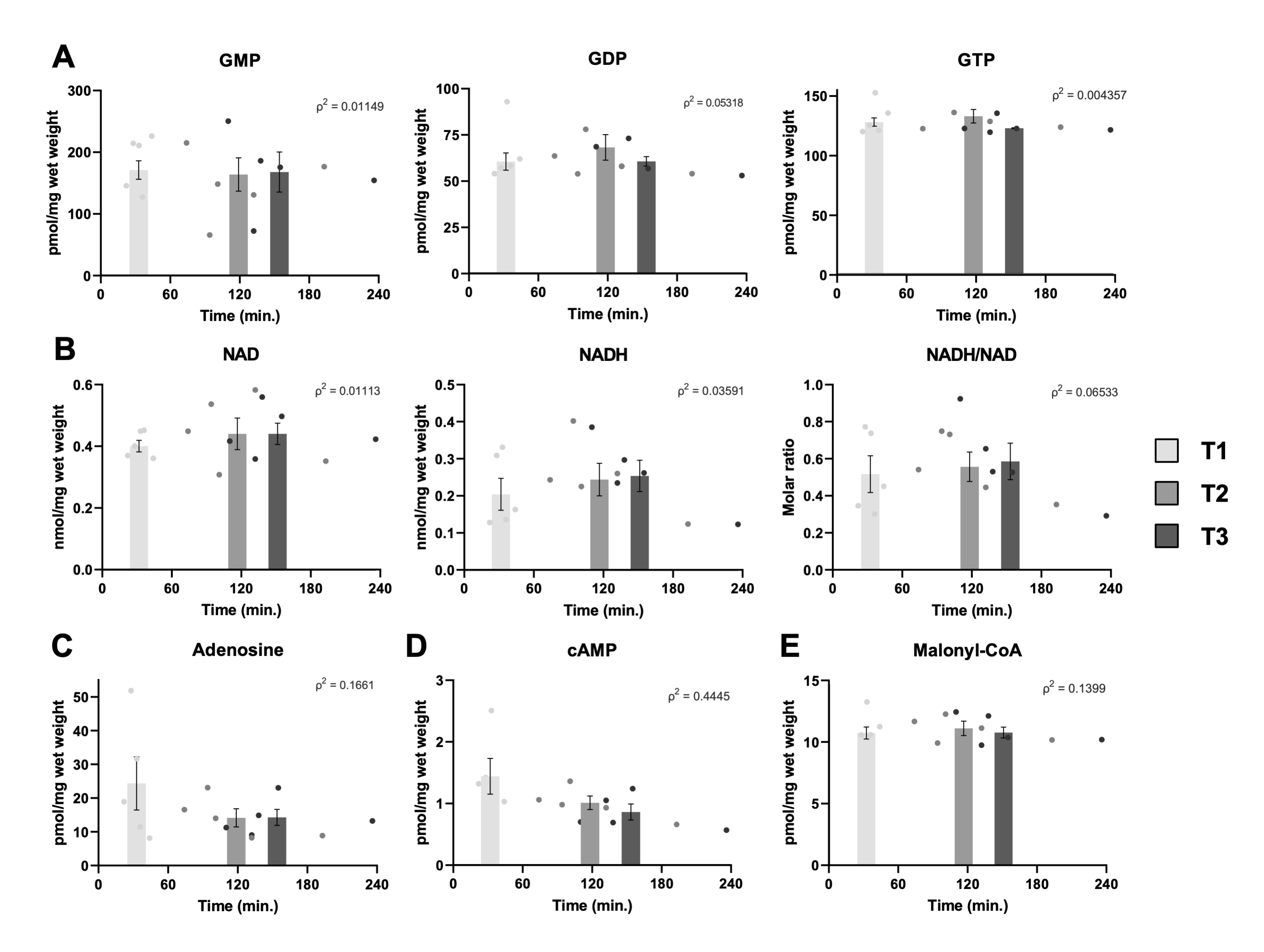

Supplement: S4 Fig — GMP, GDP, GTP (A), NAD, NADH, NADH/NAD ratio (B), adenosine (C), cyclic AMP (cAMP (D)), and malonyl-CoA (E) were quantified in collected liver samples using LC-MS targeted metabolomics analyses. (TIFF) [file pone.0304405.s004.tiff]
